# Fatal Emphysematous Tonsillopharyngitis With Laryngocele Involvement Revealing Acute Myeloid Leukemia M5 Subtype: A Previously Undescribed Presentation

**DOI:** 10.1155/crom/7278748

**Published:** 2026-05-05

**Authors:** Somaya Al Kiswani, Mohammed Abduljabbar Abed, Hossam Salameh, Muna Shahwan, Omar Sawafta, Suhaib Eid, Fatena A. H. Ajlouni, Abdullah Nofal

**Affiliations:** ^1^ Radiology Department, King Hussein Cancer Center, Amman, Jordan, khcc.jo; ^2^ Department of Medicine, An-Najah National University, Nablus, State of Palestine, najah.edu; ^3^ ENT Department, Arab Medical Center, Amman, Jordan

**Keywords:** acute myeloid leukemia, airway compromise, computed tomography, emphysematous tonsillopharyngitis, extramedullary leukemic infiltration, gas-forming infection, laryngocele

## Abstract

Emphysematous infections in the tonsillopharyngeal area are exceptionally unique; this type of infection occurs in immunocompromised patients. Extramedullary disease can occur in acute myeloid leukemia (AML), but oropharyngeal involvement is rare and diagnostically challenging. Furthermore, a laryngocele is defined as an abnormal dilation of the laryngeal saccule that is filled with air and fluid if infected. Although infections and cancers rarely extend to the laryngocele, they can be found in patients with laryngeal or hypopharyngeal cancer. We describe a 65‐year‐old male presenting with progressive sore throat, halitosis, and airway compromise. Contrast‐enhanced computed tomography demonstrated bilateral emphysematous tonsillitis with parapharyngeal extension, severe supraglottic narrowing, and rare secondary involvement of a laryngocele. Histopathology demonstrated diffuse infiltrates of atypical mononuclear cells with a high nuclear‐to‐cytoplasmic ratio, irregular chromatin, and prominent nucleoli. Immunohistochemistry showed positivity for CD45, CD33, CD68, and MPO, confirming extramedullary AML‐M5 infiltration of the tonsils. This case illustrates a rare radiologic and oncologic occurrence and the necessity for developing a high index of suspicion for identifying atypical sources of gas‐forming infections in the head‐and‐neck area that may result from a malignancy; consequently, prompt diagnosis and intervention should take place.

## 1. Introduction

Acute myeloid leukemia (AML) is characterized by abnormal proliferation of immature myeloid cells in the hematologic system [1]. Although bone marrow involvement is the primary source of disease manifestation, AML may also present in extramedullary sites as myeloid sarcoma, commonly affecting the skin, lymph nodes, bones, and central nervous system (CNS) [2]. Upper aerodigestive tract involvement is rare, and oropharyngeal infiltration is extremely uncommon [3]. When present, distinguishing symptoms of AML from severe tonsillopharyngeal infection may be challenging, potentially delaying diagnosis and leading to rapid clinical deterioration in newly diagnosed or untreated patients [4].

Emphysematous infections producing gas within the soft tissues of the neck and face are exceedingly rare but potentially life‐threatening [5]. These infections most frequently occur in immunocompromised patients, including those with diabetes mellitus, malignancy, or other systemic conditions [6]. Early clinical signs may be subtle, but these infections can quickly progress to tissue necrosis, sepsis, and airway compromise. Contrast‐enhanced CT imaging remains the diagnostic modality of choice, enabling detection of gas within tissues, assessment of deep neck space involvement, and evaluation of airway narrowing [7].

Although very few cases of emphysematous tonsillitis have been reported, totaling only 9 cases of patients according to a recent systematic search in a review published in 2024 by Moffatt et al. [8], there is a paucity of literature addressing the presentation of emphysematous tonsillitis, extramedullary AML infiltration, and rapid progression to airway compromise.

This case demonstrates a unique sequence of events culminating in a fatal outcome, emphasizing the importance of considering atypical gas‐forming neck infections as potential manifestations of malignant infiltration in immunocompromised patients.

## 2. Case Presentation

A 65‐year‐old nonsmoking male presented with a 10‐day history of progressively worsening sore throat associated with decreased oral intake and marked halitosis in the emergency department. He had received oral antibiotics at an outside facility without improvement. There were no complaints of fever, respiratory symptoms, weight loss, or constitutional symptoms. He denied voice changes such as a “hot‐potato” voice, which can be associated with upper airway obstruction.

Upon evaluation in the emergency department, his vital signs were as follows: temperature of 37.8°C, heart rate of 85 beats per minute, respiratory rate of 16 breaths per minute, blood pressure of 155/82 mmHg, and oxygen saturation of 95% on room air. On physical examination, the patient was conscious, alert, and oriented to person, place, and time. Signs of increased work of breathing were present, including nasal flaring, along with marked halitosis. Oropharyngeal examination revealed a bulky oral cavity and erythematous pharyngeal mucosa. The extremities were covered in scattered bruises.

Initial laboratory evaluation revealed leukocytosis (WBC 42 × 10^9^/L), thrombocytopenia (platelets 38 × 10^9^/L), markedly elevated inflammatory markers (CRP 156 mg/L), and mildly prolonged coagulation profile (INR 1.6). The diagnosis of AML (AML‐M5) was confirmed by peripheral blood flow cytometry.

**Figure 1:**
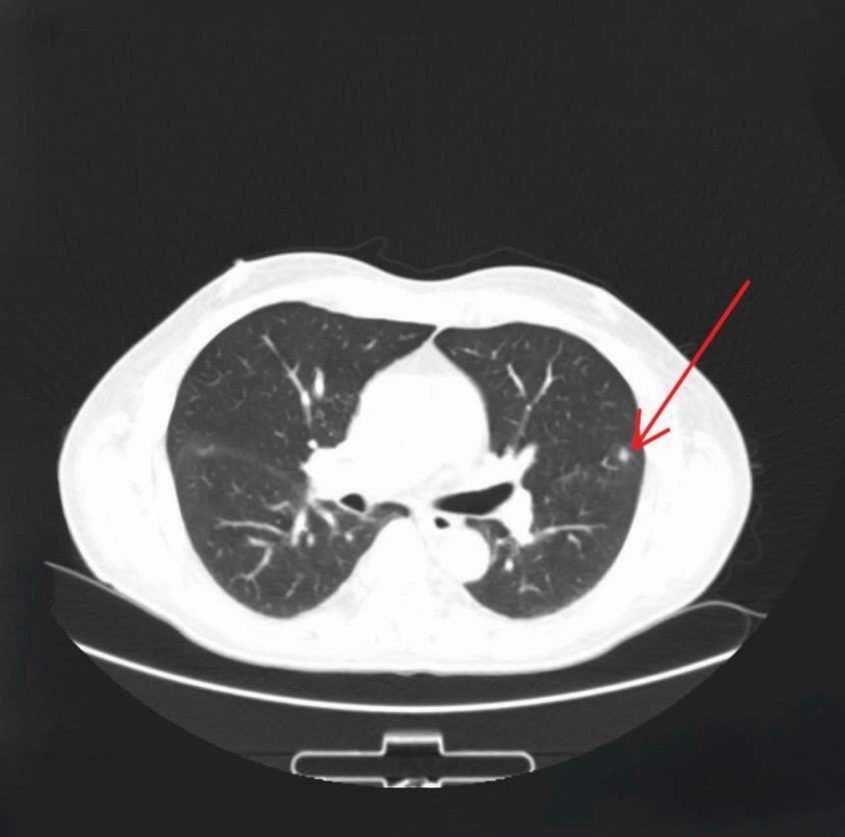
Contrast‐enhanced CT of the chest demonstrating multiple bilateral consolidative nodules scattered throughout both lung fields, consistent with pulmonary involvement in the setting of acute myeloid leukemia and systemic infection.

Contrast‐enhanced computed tomography of the chest demonstrated multiple bilateral consolidative nodules involving both lung fields, concerning for infectious or leukemic pulmonary involvement (shown in Figure 1).

**Figure 2:**
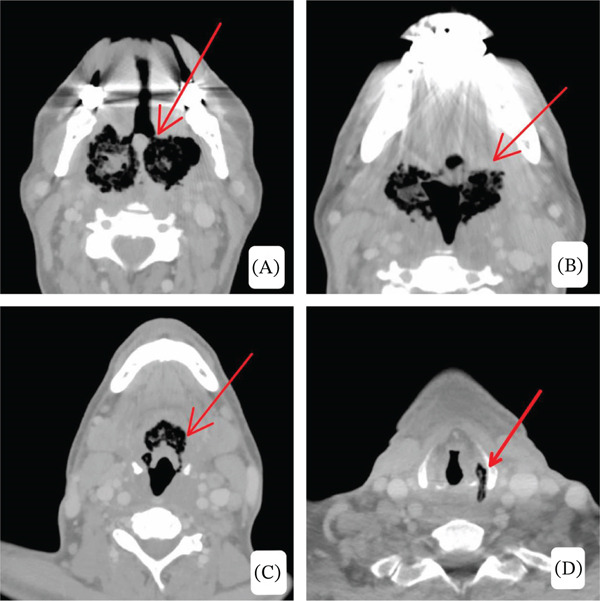
(A–D) Axial contrast‐enhanced CT images at multiple levels demonstrate central and submucosal gas locules within the palatine tonsillar parenchyma, causing diffuse almost symmetrical bilateral tonsillar enlargement and surrounding inflammatory changes, with extension into the adjacent parapharyngeal space. (C) At the periepiglottic level, there is circumferential inflammatory involvement with gas locules, resulting in significant airway narrowing. (D) At more inferior levels, there is involvement of a left‐sided laryngocele, which also contains gas and inflammatory changes, reflecting inferior extension of the same emphysematous infectious process.

**Figure 3:**
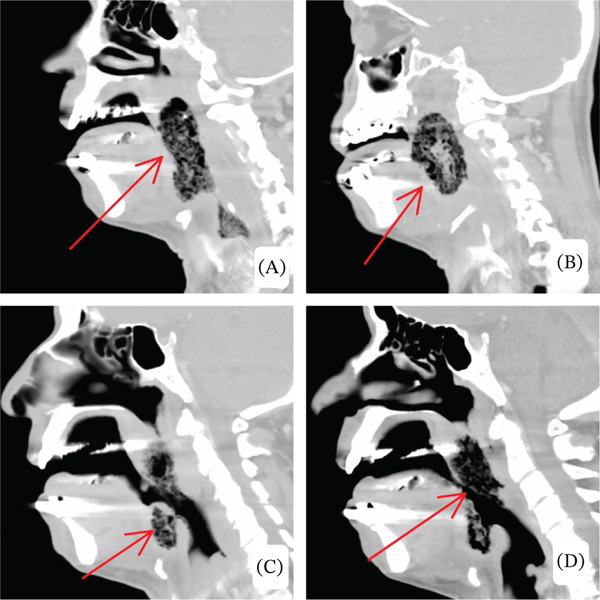
(A–D) Sagittal contrast‐enhanced CT images at multiple levels demonstrate diffuse tonsillar enlargement with central and submucosal gas locules, associated with extensive surrounding inflammatory changes, representing the same emphysematous tonsillar infection seen on axial images. (C) At the periepiglottic level, there is circumferential inflammatory involvement with gas locules, resulting in marked airway narrowing.

**Figure 4:**
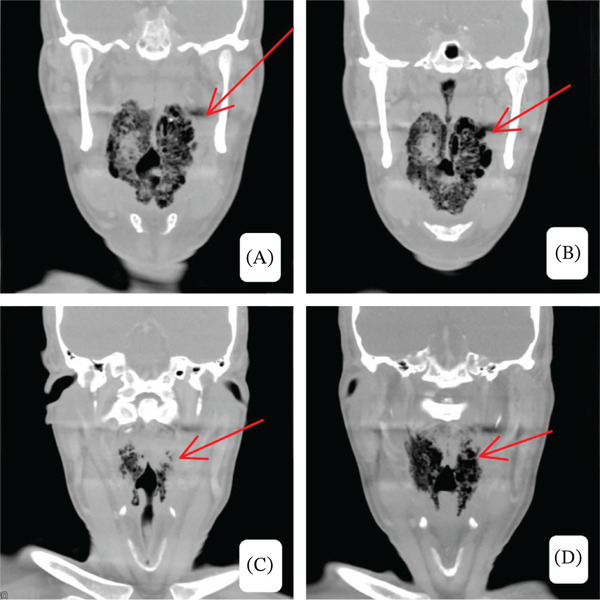
(A–D) Coronal contrast‐enhanced CT images at multiple levels demonstrate central and submucosal gas locules within the palatine tonsillar parenchyma, diffuse tonsillar enlargement, and surrounding inflammatory changes, consistent with emphysematous tonsillar infection. There is severe airway narrowing reflecting the critical compromise of the upper airway.

Given concern for deep neck space infection and potential airway compromise, contrast‐enhanced CT demonstrated diffuse bilateral tonsillar enlargement with intraparenchymal gas and surrounding inflammatory changes extending into the parapharyngeal spaces, associated with significant supraglottic airway narrowing. Imaging findings were reviewed by head‐and‐neck radiology and classified as an infected combined laryngocele with secondary inflammatory extension rather than primary laryngocele pathology (shown in Figures 2, 3, and 4).

The otolaryngology team was consulted and recommended ICU admission for close monitoring, correction of coagulopathy, and initiation of broad‐spectrum antimicrobial therapy. Awake fiberoptic nasotracheal intubation was selected due to anticipated difficult airway, distorted anatomy, progressive supraglottic narrowing, and high risk of complete obstruction with sedation‐assisted intubation.

Despite antimicrobial therapy, the patient′s condition deteriorated. Operative endoscopic evaluation by ENT revealed severe palatal and lateral pharyngeal edema with near–airway obstruction and extensive mucosal necrosis, without a drainable abscess. Biopsies were taken from necrotic areas of the left tonsil and right tongue base, and a Fucidin‐soaked pharyngeal pack was placed.

**Figure 5:**
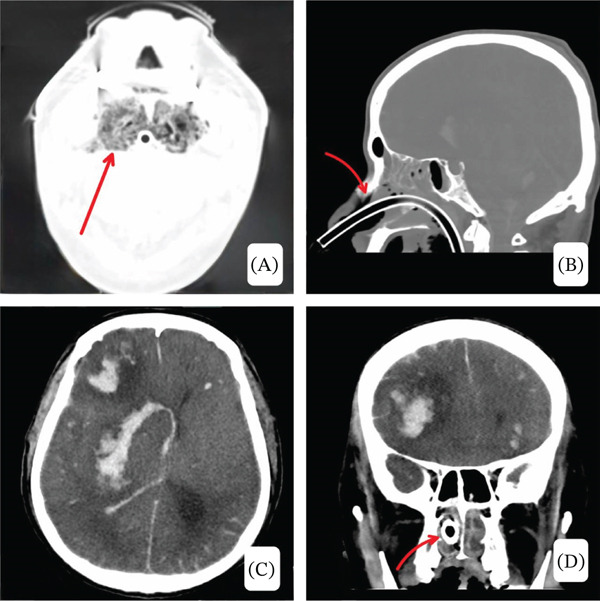
(A–D) (A) Follow‐up CT images demonstrate persistent emphysematous tonsillitis with central and submucosal gas locules, diffuse tonsillar enlargement, and surrounding inflammatory changes. (B) The paranasal sinuses show partial opacification with air‐fluid levels, and the nasogastric tube is clearly visible. (C, D) Multiple intraparenchymal hemorrhages are present supratentorially and infratentorially, with surrounding edema, mass effect, midline shift (~1.3 cm), subfalcine and uncal herniation, and cerebellar tonsillar herniation (~1.1 cm), along with diffuse subarachnoid and mild‐to‐moderate intraventricular hemorrhage and an area of hypoattenuation in the right occipital region. (D) The nasogastric tube is also visible on the coronal image.

Cultures from tracheal aspirate and tissue grew *Pseudomonas aeruginosa*, *Klebsiella oxytoca*, and *Aeromonas caviae* complex. Antimicrobial susceptibility testing demonstrated multidrug resistance but preserved sensitivity to ceftazidime–avibactam and aztreonam, guiding escalation of therapy. Histopathology of the tonsillar biopsy demonstrated leukemic infiltration, confirming extramedullary AML. Follow‐up computed tomography demonstrated persistent emphysematous tonsillitis with progressive inflammatory changes, as well as catastrophic intracranial hemorrhagic complications (shown in Figure 5). Immunohistochemistry showed positivity for CD45, CD33, CD68, and MPO, confirming extramedullary AML‐M5 infiltration of the tonsils.

Bone marrow biopsy confirmed AML‐M5. The patient received cytarabine for cytoreduction followed by standard 7 + 3 induction chemotherapy. High‐dose corticosteroids were administered for airway edema. He remained deeply sedated, nasally intubated, and mechanically ventilated, with tracheostomy planned pending correction of coagulopathy. Despite aggressive multidisciplinary management, the patient′s condition continued to deteriorate, and he unfortunately died 1 week later due to refractory septic shock. (Table 1).

**Table 1:** Clinical timeline and key events.

Day	Key events and findings
10 days before admission	Onset of sore throat, halitosis, and decreased oral intake.
7–1 days before admission	Oral antibiotics at an outside facility without clinical improvement.
Day of admission (ED/ICU)	Mild fever (37.8°C) and respiratory distress. Labs: leukocytosis, thrombocytopenia, ↑CRP, ↑INR; flow cytometry consistent with AML. CT showed bilateral consolidations and emphysematous tonsillitis with parapharyngeal extension. IV antibiotics started; awake fiberoptic intubation performed.
Day 1	ENT endoscopy showed severe edema and necrosis without abscess formation; biopsies obtained.
Day 2	Cultures grew Pseudomonas aeruginosa, Klebsiella oxytoca, and Aeromonas caviae; antibiotics escalated accordingly. Histopathology confirmed extramedullary AML.
Day 3	CT imaging demonstrated persistent emphysematous infection with radiologic progression.
Days 3–4	Bone marrow examination confirmed AML‐M5.
Day 4	Cytarabine cytoreduction followed by induction chemotherapy was initiated.
Days 5–6	Progressive clinical deterioration despite antimicrobial and oncologic treatment.
Day 7	Death secondary to refractory septic shock.

## 3. Discussion

Although emphysematous infections occur more commonly in other body areas, head and neck region infections are exceptionally rare [8]. Rather than abscess formation with other deep neck infections, emphysematous infections involve organism‐produced gas and associated tissue necrosis; in contrast, they manifest radiologically as gas‐containing spaces without associated perforations or recent instrumental manipulation [8].

Laryngocele formation in leukemia may result from multiple interacting mechanisms. These include direct leukemic infiltration of laryngeal tissues, leading to structural weakening; obstruction of the laryngeal saccule due to inflammation, edema, or necrotic changes; and external compression from infiltrated tissues or lymphoid enlargement, impairing normal saccular drainage [9, 10].

In this case, biopsy‐proven mucosal leukemic infiltration and severe inflammatory/necrotic airway changes were the most likely contributors, promoting obstruction and subsequent air entrapment, resulting in secondary laryngocele formation.

A focused literature review identified only a limited number of reported emphysematous tonsillitis cases worldwide and none describing concurrent extramedullary AML infiltration with secondary laryngocele involvement. This combination of findings therefore appears to represent a previously undescribed clinicoradiologic entity.

Although immunocompromised patients are at risk of unusual infections such as emphysematous upper aerodigestive tract infections, immunocompetent patients are not exempt from such conditions [11].

Although the pathogenesis of emphysematous tonsillitis is multifactorial, the host′s compromised immune status renders the patient vulnerable to all sorts of infections [12].

AML is described as a clonal proliferation of immature myeloid‐derived components expanding in the bone marrow as blasts and manifesting clinically as a multisystemic process such as a combination of fatigue, pallor, and organomegaly due to ineffective erythropoiesis [13].

Due to the bone marrow involvement, AML patients are considered immunocompromised, necessitating the need for an organized multidisciplinary cooperative approach including educating the patient on the status and how to avoid and reduce risks of potentially being exposed to various infectious agents, including vaccination, lifestyle, and regular follow‐up [13].

The coexistence of emphysematous infection and presence of AML‐related immunocompromise, with rapid progression into airway compromise and secondary extension into a laryngocele represents the constellation of findings that, to the best of our knowledge, has never been documented before.

This case highlights an important diagnostic principle: Atypical gas‐forming infections of the upper aerodigestive tract should prompt evaluation for underlying hematologic malignancy, particularly when accompanied by cytopenias or abnormal leukocyte counts. Early recognition may alter airway planning, antimicrobial selection, and oncologic management.

## 4. Conclusion

The present case illustrates an exceptionally rare and fulminant presentation of a sequela of AML‐related emphysematous tonsillitis with rapid progression into a life‐threatening laryngocele, emphasizing the immunocompromised sequela of AML‐related and expanding the spectrum of extramedullary AML manifestations in cases of severe deep head and neck infections.

## Funding

No funding was received for this manuscript.

## Disclosure

The authors have nothing to report.

## Ethics Statement

The authors obtained verbal and written informed consent from the patient regarding this case and any accompanying images. A copy of the written consent is available for review by the editor in chief of this journal on request. The authors declare compliance with ethical standards. All procedures performed in this report involving human participants were in accordance with the ethical standards of the institutional, national research committee, and with the 1964 Helsinki declaration and its later amendments or comparable ethical standards.

## Consent

The institution to which this case was admitted does not require approval for writing this case report.

## Conflicts of Interest

The authors declare no conflicts of interest.

## Data Availability

Data sharing is not applicable—no new data generated, or the article describes entirely theoretical research.

## References

[1] Döhner H. , Estey E. , Grimwade D. , Amadori S. , Appelbaum F. R. , Büchner T. , Dombret H. , Ebert B. L. , Fenaux P. , Larson R. A. , Levine R. L. , Lo-Coco F. , Naoe T. , Niederwieser D. , Ossenkoppele G. J. , Sanz M. , Sierra J. , Tallman M. S. , Tien H. F. , Wei A. H. , Löwenberg B. , and Bloomfield C. D. , Diagnosis and Management of AML in Adults: 2017 ELN Recommendations From an International Expert Panel, Blood. (2017) 129, no. 4, 424–447, 10.1182/blood-2016-08-733196, 2-s2.0-85014879329, 27895058. 27895058 PMC5291965

[2] Pileri S. A. , Ascani S. , Cox M. C. , Campidelli C. , Bacci F. , Piccioli M. , Piccaluga P. P. , Agostinelli C. , Asioli S. , Novero D. , Bisceglia M. , Ponzoni M. , Gentile A. , Rinaldi P. , Franco V. , Vincelli D. , Jr A. P. , Gasbarra R. , Falini B. , Zinzani P. L. , and Baccarani M. , Myeloid Sarcoma: Clinico-Pathologic, Phenotypic and Cytogenetic Analysis of 92 Adult Patients, Leukemia. (2007) 21, no. 2, 340–350, 10.1038/sj.leu.2404491, 2-s2.0-33846524299, 17170724. 17170724

[3] Mobashir M. K. , Basha W. M. , Mohamed A. E. S. , Hassaan M. , and Anany A. M. , Laryngoceles: Concepts of Diagnosis and Management, Ear, Nose & Throat Journal. (2017) 96, no. 3, 133–138, 10.1177/014556131709600313, 28346644. 28346644

[4] Bazaadut S. , Soodin D. , Singh P. , Khalafallah A. , Withers S. , Taylor S. , and Fernando R. , Extramedullary Plasmacytoma of the Tonsil With Nodal Involvement, International Journal of Otolaryngology. (2010) 2010, 302656, 10.1155/2010/302656, 20706681. 20706681 PMC2913787

[5] Brook I. , Microbiology and Antimicrobial Management of Head and Neck Infections in Children, Advances in Pediatrics. (2008) 55, no. 1, 305–325, 10.1016/j.yapd.2008.07.002, 2-s2.0-52049083236. 19048735

[6] Aydin A. , Revend L. , Revend D. , Schuck O. , and Dudde F. , Necrotizing Fasciitis of the Head and Neck – Clinical Features, Diagnostics, and Management Strategies, Oncoscience. (2025) 12, no. 12, 219–225, 10.18632/oncoscience.639. 41446318 PMC12723960

[7] Hirvonen J. , Lingam R. K. , and Connor S. , ESR Essentials: Acute Infections of the Head and Neck-Practice Recommendations by the European Society of Head and Neck Radiology, European Radiology. (2026) 36, no. 1, 334–343, 10.1007/s00330-025-11818-4, 40702317. 40702317 PMC12712113

[8] Moffatt C. , Maldonado S. T. , Evans L. K. , Azizyan A. , and Blackwell K. E. , Mucosal Emphysematous Head and Neck Infections: Scoping Review and a Case of Emphysematous Tonsillitis, Laryngoscope Investigative Otolaryngology. (2024) 9, no. 3, e1274, 10.1002/lio2.1274, 38803461. 38803461 PMC11129548

[9] Juneja R. , Arora N. , Meher R. , Mittal P. , Passey J. C. , Saxena A. , and Bhargava E. K. , Laryngocele: A Rare Case Report and Review of Literature, Indian Journal of Otolaryngology and Head & Neck Surgery. (2019) 71, no. 1, 147–151, 10.1007/s12070-017-1162-x, 2-s2.0-85025446510, 31741950. 31741950 PMC6848569

[10] Singh R. , Karantanis W. , Fadhil M. , Kumar S. A. , Crawford J. , and Jacobson I. , Systematic Review of Laryngocele and Pyolaryngocele Management in the Age of Robotic Surgery, Journal of International Medical Research. (2020) 48, no. 10, 300060520940441, 10.1177/0300060520940441. 33100073 PMC7604991

[11] Sunyecz I. , Orabi N. , and Coutras S. , Acute Emphysematous Epiglottitis: A Case Report, Laryngoscope. (2023) 133, no. 10, 2747–2750, 10.1002/lary.30660, 36929847. 36929847

[12] Thakur J. S. , Mohindroo N. K. , Sharma D. R. , Mohindroo S. , and Thakur A. , Tonsillitis With Acute Myeloid Leukemia: A Case Series for Caution, Ear, Nose, & Throat Journal. (2013) 92, no. 4, E22–E23, 10.1177/014556131309200425, 23599112. 23599112

[13] Vakiti A. , Reynolds S. B. , and Mewawalla P. , Acute Myeloid Leukemia, StatPearls, 2025, StatPearls Publishing, http://www.ncbi.nlm.nih.gov/books/NBK507875/. 29939652

